# Geometric Nonlinear Analysis of Self-Anchored Cable-Stayed Suspension Bridges

**DOI:** 10.1155/2013/734387

**Published:** 2013-10-24

**Authors:** Wang Hui-Li, Tan Yan-Bin, Qin Si-Feng, Zhang Zhe

**Affiliations:** ^1^Bridge Engineering Research Institute, Dalian University of Technology, Dalian 116085, China; ^2^Research Center for Numerical Tests on Material Failure, Dalian University, Dalian 116622, China

## Abstract

Geometric nonlinearity of self-anchored cable-stayed suspension bridges is studied in this paper. The repercussion of shrinkage and creep of concrete, rise-to-span ratio, and girder camber on the system is discussed. A self-anchored cable-stayed suspension bridge with a main span of 800 m is analyzed with linear theory, second-order theory, and nonlinear theory, respectively. In the condition of various rise-to-span ratios and girder cambers, the moments and displacements of both the girder and the pylon under live load are acquired. Based on the results it is derived that the second-order theory can be adopted to analyze a self-anchored cable-stayed suspension bridge with a main span of 800 m, and the error is less than 6%. The shrinkage and creep of concrete impose a conspicuous impact on the structure. And it outmatches suspension bridges for system stiffness. As the rise-to-span ratio increases, the axial forces of the main cable and the girder decline. The system stiffness rises with the girder camber being employed.

## 1. Introduction

The self-anchored cable-stayed suspension bridge dates back to the early 19th century [1, 2]. First a mixed structure of cable-stayed and suspension bridge was built in France. After years of trial and effort, it has evolved to Roebling system, Dichinger system, and then the improved Dichinger system. The suspended part of the self-anchored cable-stayed suspension bridge is much shorter than that of suspension bridge with the same overall span; therefore the main cable force can be reduced in a large extent. What is more, the cantilever length of cable-stayed part can be decreased greatly during the construction period, thereby improving aerodynamic stability of the structure. This bridge system has been proposed for many design projects, such as the Strait of Gibraltar Bridge project and the Lingdingyang Bridge project [3].

Until now the cable-stayed suspension bridge still remains in the design proposal phase. No large-span cable-stayed suspension bridge has ever been built in the world. And all the design proposals are earth-anchored systems. Both the anchorage and the construction period can be saved through adopting the self-anchored system. The self-anchored cable-stayed suspension bridge suits the long-span needs well. Long-span cable-stayed bridges, suspension bridges, and cable-stayed suspension bridges have been discussed a lot, but there are few papers relating to self-anchored cable-stayed suspension bridges [4]. Based on a self-anchored cable-stayed suspension bridge with an 800 m main span, its geometric nonlinearity under live load is studied.

## 2. The Geometric Nonlinear Characteristics of the Cooperation System

Three main factors cause the geometric nonlinearity of the cooperation system, including the cable sag, large displacements, and the initial internal force.

### 2.1. The Cable Sag Effect

The cable would sag in the free suspension state. The sag and the chord length of the cable change as the internal force alters. A nonlinear relationship exists between the chord length and the cable force. The method of equivalent elasticity modulus can be adopted to simulate the sag effect. The well-known Ernst formula for equivalent elasticity modulus is shown as follows:

(1) $$\begin{array}{c} E_{\text{eq}}=\frac{E}{\left[1+w^{2}L^{2}AE/\left(12T^{3}\right)\right]}, \end{array}$$

where *E* indicates the elasticity modulus of the cable, *L* the horizontal projection length of the cable element, *w* the cable weight per unit length, *A* the cross-sectional area of the cable, and *T* the axial force of the cable element. The cable element can be set up as a straight bar with the elastic modulus correction.

### 2.2. The Effect of the Initial Internal Force and Large Displacements

The girder and main pylon of the cooperation system bear tremendous pressure. The axial force causes additional bending moments, which affects the bending stiffness of components; meanwhile the bending moment alters the length of structure components, which furthermore affects the axial stiffness of components. By introducing the initial stress stiffness matrix [*K*]_*G*_ to simulate the effect of the initial internal force and stiffness matrix of displacement [*K*]_*L*_ to model the effect of large displacements, the initial tangent stiffness matrix can be attained [5]:

(2) $$\begin{array}{c} \left[K\right]={\left[K\right]}_{0}+{\left[K\right]}_{G}+{\left[K\right]}_{L}, \end{array}$$

where [*K*]_0_ represents linear stiffness matrix.

### 2.3. Second-Order Analysis Theory

Geometric nonlinearity leads to the failure in the application of superposition principles. The moment and displacement caused by live load cannot be calculated with influence lines. The response of the cooperation system under all kinds of loads can be attained with the method described above. However, it is time-consuming, especially for the live load which requires repeated iterations. Second-order theory, a simplified method of approximate calculation, will be discussed as follows.

The girder and pylon of the cooperation system belong to compression-bending members. A simply planar differential equation for compression bending beams is shown below [6]:

(3) $$\begin{array}{c} EI\nu^{\left(4\right)}+N\left(x\right)\nu^{\left(2\right)}=q\left(x\right). \end{array}$$

The axial force *N*(*x*) of the cooperation system consists of two parts. The one caused by the dead load is recorded as *N*_*g*_(*x*); the other one caused by live load is denoted by *N*_*q*_(*x*). Therefore, (3) is a nonlinear differential equation.

Dr. Li Guohao solved the second-order nonlinear catenaries theory with linear method [7]. By drawing on this idea, the nonlinear analysis of the cooperation system can be simplified. The proportion of live load to dead load for long-span bridges usually ranges from 10% to 20% [8]. Therefore the effect of *N*_*q*_(*x*) is negligible. Then (3) can be simplified into

(4) $$\begin{array}{c} EI\nu^{\left(4\right)}+N_{g}\left(x\right)\nu^{\left(2\right)}=q\left(x\right). \end{array}$$

Equation (4) is a linear differential equation, which satisfies the condition of linear superposition. This simplified method can be called second-order theory [9].

## 3. Case Study

**Figure 1:**
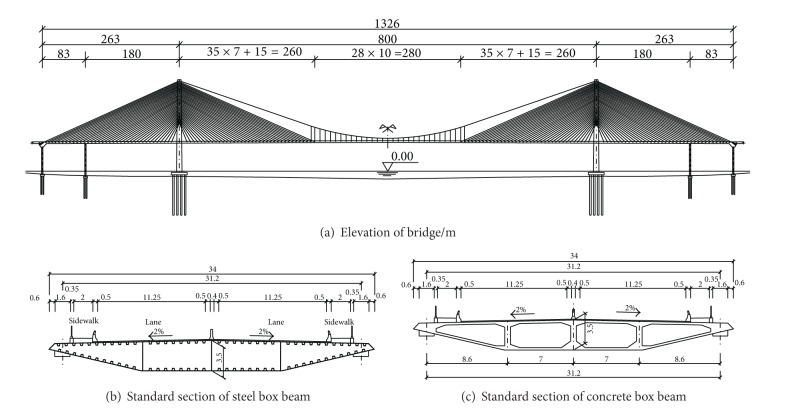
Arrangement diagram of Dalian Gulf Bridge/m.

The Dalian Gulf Bridge with a main span of 800 m, is in the form of a self-anchored cable-stayed suspension bridge. Its total length is 1326 m. The main girder section adopts streamlined flat box girder, which is 3.5 m high and 34 m wide. There are two kinds of main girder for the whole bridge including steel girder and prestressed concrete girder. The steel part is in the middle suspension segment and the prestressed concrete part in the cable-stayed area. The H-shaped pylon is 127 m above the main girder. The elevation layout of the bridge is shown in Figure 1.

### 3.1. Study on the Nonlinearity of Live Load

**Figure 2:**
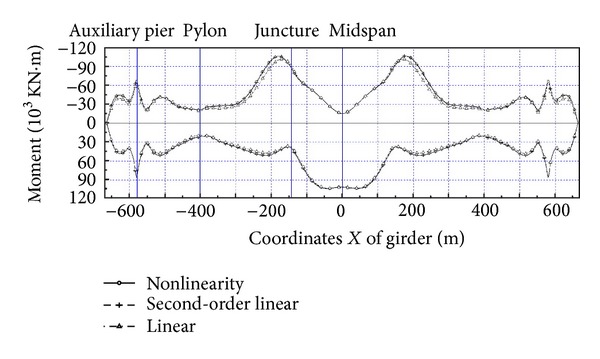
Envelope of main girder moment.

**Figure 3:**
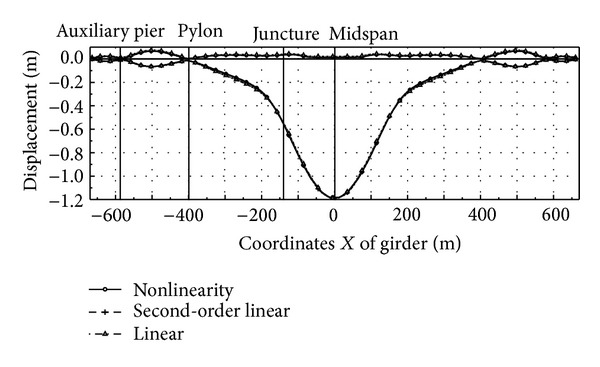
Envelope of main girder displacement.

**Figure 4:**
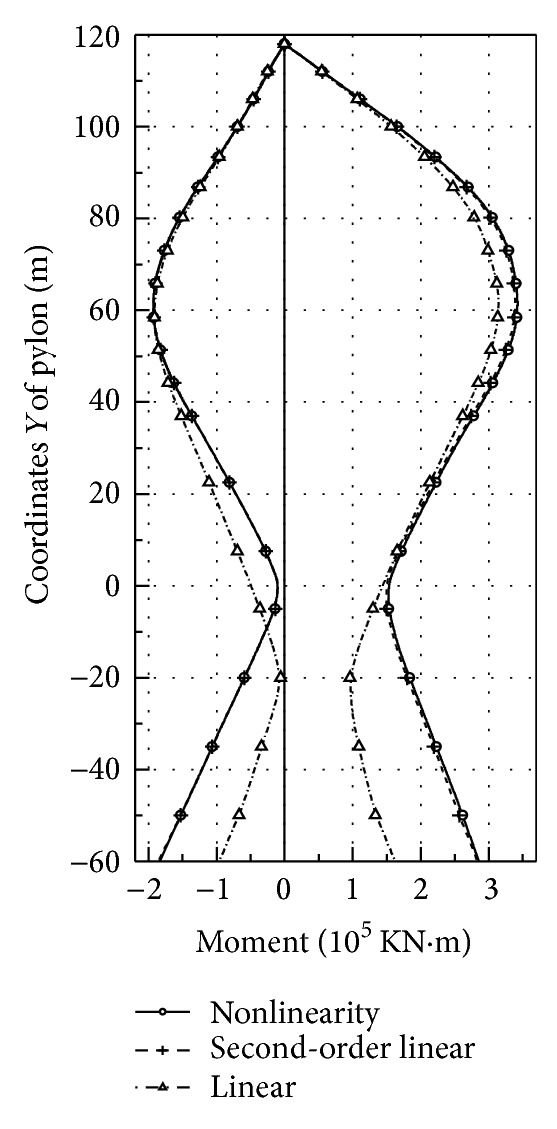
Envelope of pylon moment.

**Figure 5:**
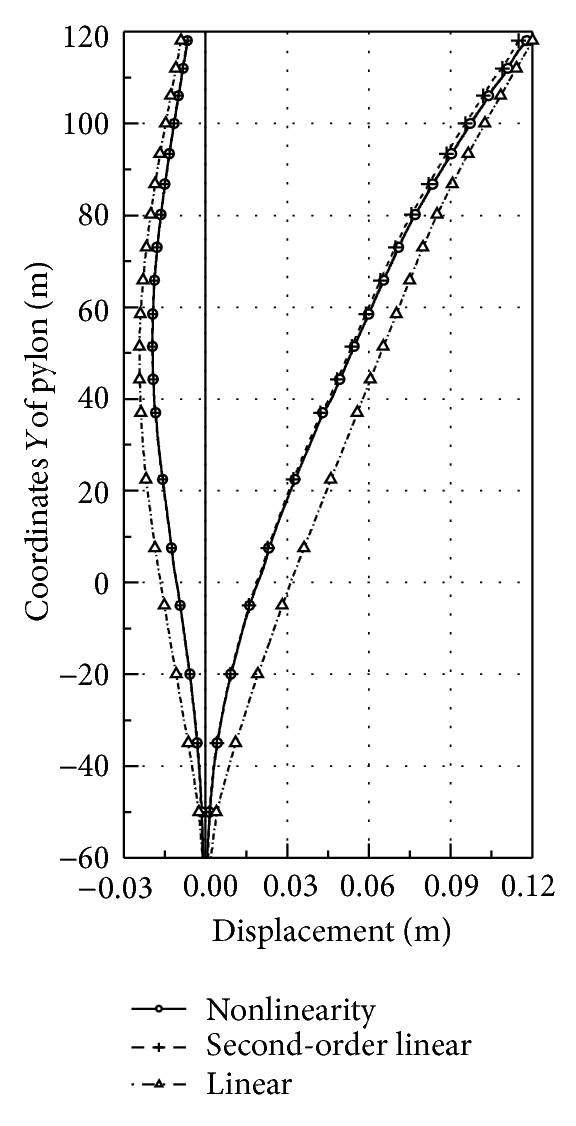
Envelope of pylon displacement (positive direction pointing to mid-span).

Here the nonlinearity of live load is analyzed with nonlinear theory, second-order theory, and linear theory, respectively. Since the superposition principle becomes inappropriate in the nonlinear analysis, the influence zone method is adopted. Under the automobile load of grade I (China), the moment and displacement envelope diagram of main girder and main pylon are as shown in Figures 2, 3, 4, and 5. The results of these calculations are summarized in Table 1.

1. The result of linear theory radically differs from that of nonlinear theory, while the result of second-order theory is close to that of nonlinear theory. The maximum relative difference between the results of second-order theory and nonlinear theory is less than 6%, which is acceptable for the actual project. The calculation of second-order theory is simpler than that of nonlinear theory; therefore for the cooperation system bridge with a main span of 800 m, the second-order theory is feasible to calculate live load response.
2. The maximum positive moment of the main girder occurs at the middle, while the maximum negative moment occurs near the junction. Due to the great change of stiffness and the enormous negative moment, the junction between steel girder and prestressed concrete girder has to be strengthened specifically.
3. The maximum deflection at the middle of the girder is 1.18 m, which is about 1/650 of the middle span length. Therefore, the integral stiffness of the cooperation system bridge is higher than that of normal suspension bridges with the same length, due to the fact that the cable-stayed part enhances the global stiffness.

### 3.2. Study on Concrete Shrinkage and Creep

Shrinkage and creep of concrete can make girder and pylon shorter, causing the main cable and stayed-cable sag, so the bending moment and deformation of the girder increase. Based on the Bridge Criterion (China), the concrete shrinkage and creep effects within 15 years are analyzed. The results are summarized in Table 2.

The results show that concrete shrinkage and creep have an effect on internal forces and the shape of cooperation system. Effective measures should be taken to reduce the influence, such as extending the load age of concrete, using microexpansion concrete and so on. 

### 3.3. Analysis on the Impact of Rise-to-Span Ratio

**Figure 6:**
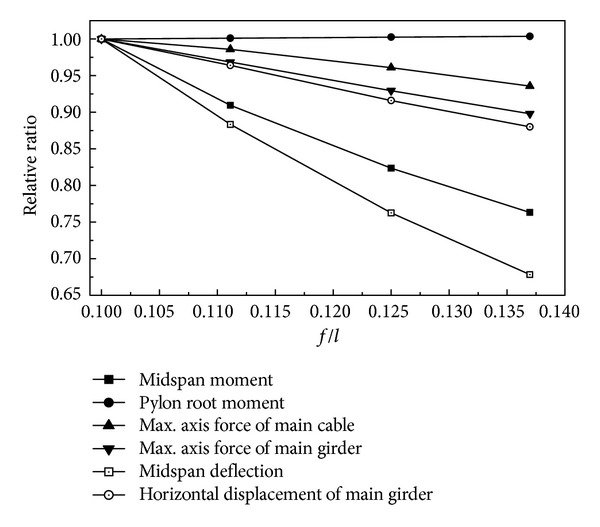
Effects of rise-span of main cable.

The rise-to-span ratio of the main cable is an important parameter for the cooperation system bridge, which affects both structural stiffness and internal forces. The impact of rise-to-span ratio under live load is given in Figure 6. For the sake of convenience, dimensionless forms are adopted. It is convenient to make the parameters dimensionless using results of rise-to-span ratio 1/10 as a reference.

Figure 6 shows that global stiffness increases as the rise-to-span ratio rises, which is the same as the cooperation system bridge but opposite to earth-anchored suspension bridges [10]. The axial forces of the girder and the main cable descend as the rise-to-span ratio increases. The axial force of the girder is extremely sensitive to the rise-to-span ratio. The axial forces of the girder and the main cable are enormous, which affects the cross-sectional areas of the girder and the main cable. So the smaller rise-to-span ratio is not recommended for the cooperation system bridge.

Figure 6 also shows that horizontal displacements of the main girder descend as the rise-to-span ratio increases because the axial force of the girder descends as the rise-to-span ratio increases. As the rise-to-span ratio increases, the moment at the pylon root rises a little.

### 3.4. Analysis on Camber of the Main Girder

Because of the high level of component force of the main cable at both ends of the main girder, the camber of the main girder will induce an additional negative bending moment for the main girder and further increase the main girder's moment due to P-Δ effect. The moment under dead load can be adjusted through the cable tension adjustment; therefore it is only necessary to analyze the main girder camber's effect under live load.  Table 3 presents the main girder's bending moments and deflections under live load with both 0 m and 2.9 m cambers at the midspan of the main girder. The results indicate that setting up a girder camber can effectively reduce the bending moment of the main girder and improve the structural stiffness.

## 4. Conclusions

Geometric nonlinear factors of the cooperation system bridge are discussed in this paper. Based on this, a cooperation system bridge with an 800 m main span is analyzed. And the following conclusions are reached.

1. The error is less than 6% using a simple second-order approximation theory to calculate live load response of a cooperation system bridge with an 800 m main span.
2. The stiffness of the junction between the cable-stayed segment and the suspension area varies. Great internal forces occur easily under live load, so it is necessary to strengthen the junction.
3. Concrete shrinkage and creep have a conspicuous impact on the internal force and deformation of the structure. It is necessary to take measures to alleviate the influence.
4. Global stiffness increases with the rise-to-span ratio ascending and the axial forces of the girder and the main cable descend as the rise-to-span ratio rises. Therefore the small rise-to-span ratios are not recommended for the cooperation system bridge.
5. Setting a girder camber can improve the integral stiffness of the cooperation system bridge.

## Figures and Tables

**Table 1:** Nonlinear effect of live load.

		Nonlinearity	Second-order	Relatively deviation%	Linear	Relatively deviation%
Moment at midspan of girder/(10^3^ KN·m)	Max	101.24	101.19	−0.05	101.7	0.45
	Min	−15.00	−14.96	−0.27	−15.04	0.27
Moment at root of pylon/(10^3^ KN·m)	Max	285.61	283.20	−0.84	162.80	−47.79
	Min	−184.12	−185.48	0.74	−96.65	−47.51
Displacement at midspan of girder/(mm)	Max	13.3	13.4	0.75	21.2	59.40
	Min	−1180	−1186	0.51	−1189	0.76
Displacement at end of girder/(mm)	Max	23.4	22.0	−5.98	21.5	−8.12
	Min	−0.84	−0.86	2.38	−0.36	−57.14
Displacement at top of pylon/(mm)	Max	118.0	115.0	−2.54	120.0	1.69
	Min	−6.6	−6.6	0.00	−9.1	37.88

**Table 2:** Effect of shrinkage and creep of concrete.

Position	Moment/(KN·m)	Displacement/(mm)
Girder		
Midspan	−358	−36.6
Supporting point at pylon	−11700	−1.0
Pylon	36150	27.2

The moment of pylon takes place at the root. The displacement of pylon takes place at top and the positive direction points to midspan. The positive direction of girder displacement points to upward side.

**Table 3:** Effect of camber of main girder.

	0 camber	2.9 m camber	Relatively deviation%
Midspan moment/(10^3^ KN·m)	101.19	99.18	−1.99
Midspan deflection/mm	1186	1151	−2.95
